# A Gene X Environment Study of a Novel Preclinical Mouse Model: The Humanized Mthfr*677C>T Allele and Inorganic Arsenic in Late‐Onset Alzheimer's Disease Progression

**DOI:** 10.1002/alz70855_106540

**Published:** 2025-12-24

**Authors:** Zeynep Sarica, Kevin P Kotredes, Alaina M Reagan, Ravi S Pandey, Adrian L Oblak, Paul R Territo, Bruce T. Lamb, Gregory W Carter, Michael Sasner, Gareth R Howell, the MODEL‐AD Consortium

**Affiliations:** ^1^ The Jackson Laboratory, Bar Harbor, ME, USA; ^2^ Stark Neuroscience Research Institute, Indianapolis, IN, USA; ^3^ Stark Neurosciences Research Institute, Indiana University School of Medicine, Indianapolis, IN, USA

## Abstract

**Background:**

Current mouse models fail to adequately capture the clinical variability and genetic complexity of late‐onset Alzheimer's disease (LOAD), limiting their utility for preclinical therapeutic development. To address this, the IU/JAX/PITT MODEL‐AD (Model Organism Development and Evaluation for Late‐Onset AD) Center is generating novel mouse strains incorporating human risk alleles associated with LOAD, aiming to better represent the disease's genetic background and phenotypic diversity. Previous studies showed that the 677C>T variant in the MTHFR gene increases susceptibility to LOAD. Additionally, environmental factors such as inorganic arsenic exposure, which has been associated with increased Alzheimer's risk, were evaluated in our model. The expression of the humanized Mthfr*C677T allele in these novel mouse strains, along with exposure to environmental insults, will deepen our understanding of LOAD and facilitate the discovery of potential biomarkers and therapeutic targets.

**Methods:**

We generated and aged LOAD2.Mthfr*677C>T (homozygous for humanized Mthfr*677C>T allele, humanized *Abeta, APOEe4*, and Trem2*R47H) and LOAD2 controls. At 23 months of age, some of the mice were exposed to 200 ppm arsenic trioxide in drinking water for 30 days, while control groups received unspiked water. Longitudinal behavioral and biometric data were collected, and tissues were analyzed at endpoints using transcriptomics, proteomics, and neuropathological approaches. Detoxification efficiency was assessed through arsenic speciation in urine using LC‐ICP‐MS, with speciation from liver and kidney samples still to be completed.

**Results:**

Reduced enzymatic activity of MTHFR*677C>T prevented homocysteine conversion, elevating blood homocysteine levels. Aged LOAD2.Mthfr*677C>T mice showed transcriptional and proteomic signatures in the brain that aligned significantly with those observed in human AD. Speciation analysis showed dimethylarsinic acid (DMA) was the major species present in the urine of treated mice, and arsenic treatment also increased alignment of transcriptional signatures to human AD. No plaques were present in LOAD2.Mthfr*677C>T but subtle changes in glial cells were observed.

**Conclusions:**

The Mthfr*677C>T variant and/or arsenic treatment modified correlation of brain transcripts or proteins to human AD, providing evidence of their utility for modeling the complexity of human LOAD.

